# The Locare workflow: representing neuroscience data locations as geometric objects in 3D brain atlases

**DOI:** 10.3389/fninf.2024.1284107

**Published:** 2024-02-09

**Authors:** Camilla H. Blixhavn, Ingrid Reiten, Heidi Kleven, Martin Øvsthus, Sharon C. Yates, Ulrike Schlegel, Maja A. Puchades, Oliver Schmid, Jan G. Bjaalie, Ingvild E. Bjerke, Trygve B. Leergaard

**Affiliations:** ^1^Neural Systems Laboratory, Department of Molecular Medicine, Institute of Basic Medical Sciences, University of Oslo, Oslo, Norway; ^2^EBRAINS AISBL, Brussels, Belgium

**Keywords:** 3D brain atlas, FAIR data, interoperability, rat brain, mouse brain, standardization, data integration

## Abstract

Neuroscientists employ a range of methods and generate increasing amounts of data describing brain structure and function. The anatomical locations from which observations or measurements originate represent a common context for data interpretation, and a starting point for identifying data of interest. However, the multimodality and abundance of brain data pose a challenge for efforts to organize, integrate, and analyze data based on anatomical locations. While structured metadata allow faceted data queries, different types of data are not easily represented in a standardized and machine-readable way that allow comparison, analysis, and queries related to anatomical relevance. To this end, three-dimensional (3D) digital brain atlases provide frameworks in which disparate multimodal and multilevel neuroscience data can be spatially represented. We propose to represent the locations of different neuroscience data as geometric objects in 3D brain atlases. Such geometric objects can be specified in a standardized file format and stored as location metadata for use with different computational tools. We here present the Locare workflow developed for defining the anatomical location of data elements from rodent brains as geometric objects. We demonstrate how the workflow can be used to define geometric objects representing multimodal and multilevel experimental neuroscience in rat or mouse brain atlases. We further propose a collection of JSON schemas (LocareJSON) for specifying geometric objects by atlas coordinates, suitable as a starting point for co-visualization of different data in an anatomical context and for enabling spatial data queries.

## 1 Introduction

Experimental brain research in animal models generates large amounts of disparate data of different modality, format, and spatial scale (Sejnowski et al., 2014). To manage and exploit the growing resource of neuroscience data it is now widely recognized that the data must be shared in accordance with the FAIR principles (Wilkinson et al., 2016), ensuring that data are findable, accessible, interoperable and reusable for future analyses (see e.g., Abrams et al., 2022). This trend has resulted in a growing volume of neuroscience data being made accessible through various data repositories and infrastructures (Ferguson et al., 2014; Jorgenson et al., 2015; Ascoli et al., 2017; Amunts et al., 2019). While free-text searches based on structured metadata are typically implemented in such databases (Clarkson, 2016), possibilities for more sophisticated queries, visualizations, and analysis depend on a harmonization across data files with different formats, scales, and organization (Zaslavsky et al., 2014; Abrams et al., 2022).

Anatomical information is widely used to provide a common context for harmonizing and comparing neuroscience data (Martone et al., 2004; Bassett and Sporns, 2017). The availability of open-access 3D rodent brain reference atlases (Oh et al., 2014; Papp et al., 2014; Wang et al., 2020; Kleven et al., 2023a) has opened up new opportunities for combining and analyzing data that have been aligned to a common spatial framework (Leergaard and Bjaalie, 2022). This allows researchers to integrate and analyze data from different sources within a common anatomical context more easily. For example, spatial registration procedures allow image data to be directly compared and analyzed based on atlas coordinates or annotated brain structures (Puchades et al., 2019; Tappan et al., 2019; Tyson and Margrie, 2022; Kleven et al., 2023b), e.g., through use of computational analyses of features of interest in atlas-defined regions of interest (Kim et al., 2017; Bjerke et al., 2018b, 2023; Yates et al., 2019; Kleven et al., 2023a,b). For other data types, such as locations of electrode tracts, 3D reconstructed neurons, or other features of interest, procedures and tools have been developed to represent the data as coordinate-based points of interest allowing validation or visualization of locations (Bjerke et al., 2018b; Fiorilli et al., 2023).

Atlases, tools, and resources for building, viewing, and using collections of spatially registered data have also proven to be fundamental for digital research infrastructures, such as the Allen Brain Map data portal^1^ and to some extent also the EBRAINS Research Infrastructure.^2^ But while the Allen institute provides collections of systematically generated homogenous and standardized image data spatially integrated in a 3D atlas, EBRAINS allows the research community to share a wide variety of data. These data may be related to anatomical locations using anatomical terms, reference to stereotaxic coordinates, or spatial registration to atlases. Thus, the location documentation provided with published data is as disparate as the data themselves—ranging from coordinate-based documentation defining the position of data in an atlas, to anatomical terms, illustrations, and unstructured descriptions (Bjerke et al., 2018a). The specification of such location metadata varies considerably, and a common standard for storing them is lacking in neuroscience. This poses a challenge to effectively utilize the metadata for spatial queries, co-visualization, and other analytic purposes. To achieve the ambitions of the community to accumulate and re-use neuroscience research data in agreement with the FAIR principles, it is necessary to represent metadata describing anatomical locations (spatial metadata) in a standardized and machine-readable format.

To address this challenge, we developed the Locare workflow (from *locãre*, latin: to place) for representing disparate neuroscience data in a simplified and standardized manner. The workflow was developed based on a large collection of diverse experimental data from mouse and rat brains shared via the EBRAINS Knowledge Graph.^3^ The available location documentation, specifying data location through points of interest, images, or semantic descriptions determines the starting point of the workflow, which through different workflow routes outputs geometric objects. We here present Locare as a generic workflow for specifying interoperable spatial metadata for neuroscience data, and exemplify how it can be used to specify anatomical locations for different data types as geometric objects in atlas space using a JSON format. The LocareJSON schemas allow representation of data in a simplified and standardized format that can enable spatial search, co-visualization, and analyses of otherwise disparate neuroscience data. The Locare workflow provides a solution for defining heterogeneous neuroscience data as atlas-defined geometric objects in a machine-readable format, which in turn can be utilized to represent data as interoperable objects in a 3D anatomical atlas and develop spatial query functionalities. The workflow is here presented in context of the EBRAINS Research Infrastructure but is generally applicable to any infrastructure of databases holding neuroscience data.

## 2 Materials and methods

The Locare workflow builds on several years of experience with assisting researchers to share and present their experimental research data through the EBRAINS Research Infrastructure. As part of this effort, we investigated how to integrate and represent rat and mouse data sets in three-dimensional (3D) brain atlases. The workflow was established using 186 mouse brain data sets and 94 rat brain data sets available from the EBRAINS Knowledge Graph by 11 May 2023. An overview of all data set titles and type of location documentation is provided in Supplementary Table 1. The data sets included data files in various formats, structured metadata, and a data descriptor including summary, materials and methods, usage notes and explanation of data records. Several data sets were also associated with journal publications containing additional images and/or textual information about the anatomical location of the data. In some cases, we were in contact with data providers (custodians of the data shared through EBRAINS) directly and received additional information. These 280 data sets were contributed by 480 different researchers and acquired using 25 different experimental methods. The anatomical locations of observations or measurements in these data sets were documented using images (*n* = 116), semantic descriptions only (*n* = 123), or by specification of coordinates for points of interest (POIs; *n* = 41).

### 2.1 Establishing the Locare workflow

**Figure 1:**
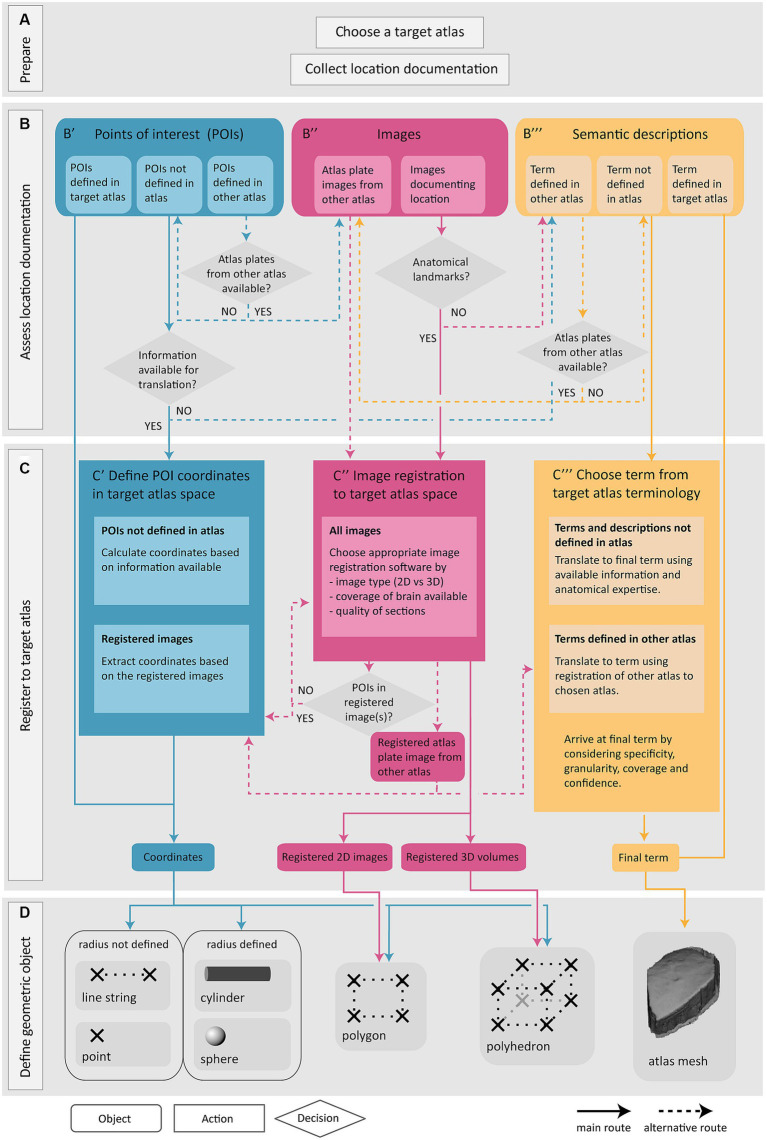
Overview of the Locare workflow. Location documentation is collected **(A)**, assessed **(B)**, and registered to a target atlas **(C)** followed by the creation of geometric objects representing the data of which the location documentation was derived **(D)**. **(A)** Preparatory steps involve choosing a target atlas in which the geometric objects should be represented and collecting of relevant location documentation. **(B)** The location documentation available, defined as points of interest (POI; **B′**), images **(B″)** or semantic descriptions **(B‴)**, determines which route of the workflow is used. **(B′,C′)** Point route: POIs may be defined in the target atlas, in another atlas, or not in an atlas. POIs defined in target atlas are directly used to create geometric objects. POIs not defined in the target atlas must be translated to coordinates of the target atlas **(C′)** (see text for details). If no information is available for translation of POIs to target atlas, the inputs are directed to semantic translation (**C‴**, blue arrow). **(B″,C″)** Image route: Images may document the location of specific data or can also be atlas plate images used to translate points of interest or semantic descriptions to a geometric object or mesh in the target atlas. Image registration is performed if possible **(C″)**, or alternatively the workflow can be directed to the semantic route (**B‴**, pink arrow). Images registered to the target atlas containing POIs may be used for coordinate extraction (**C′**, pink arrow). Atlas plate images from other atlas registered to the target atlas is used for extraction of coordinates for POIs (**C′**, pink arrow) or for translation of semantic term (**C‴**, pink arrow). **(B″,C″)** Semantic route: Semantic descriptions may be defined in the target atlas, another atlas, or not defined in an atlas. Terms defined in target atlas are directly used as the final term. Terms defined in other atlas are translated based on the spatial registration of atlas plates from the other atlas to the target atlas (**B″**, yellow arrow). Terms not defined in any atlas are translated to the most closely corresponding term in the target atlas **(C‴)**. **(D)** The output of the workflow routes is one or several geometric objects or atlas meshes.

**Figure 2:**
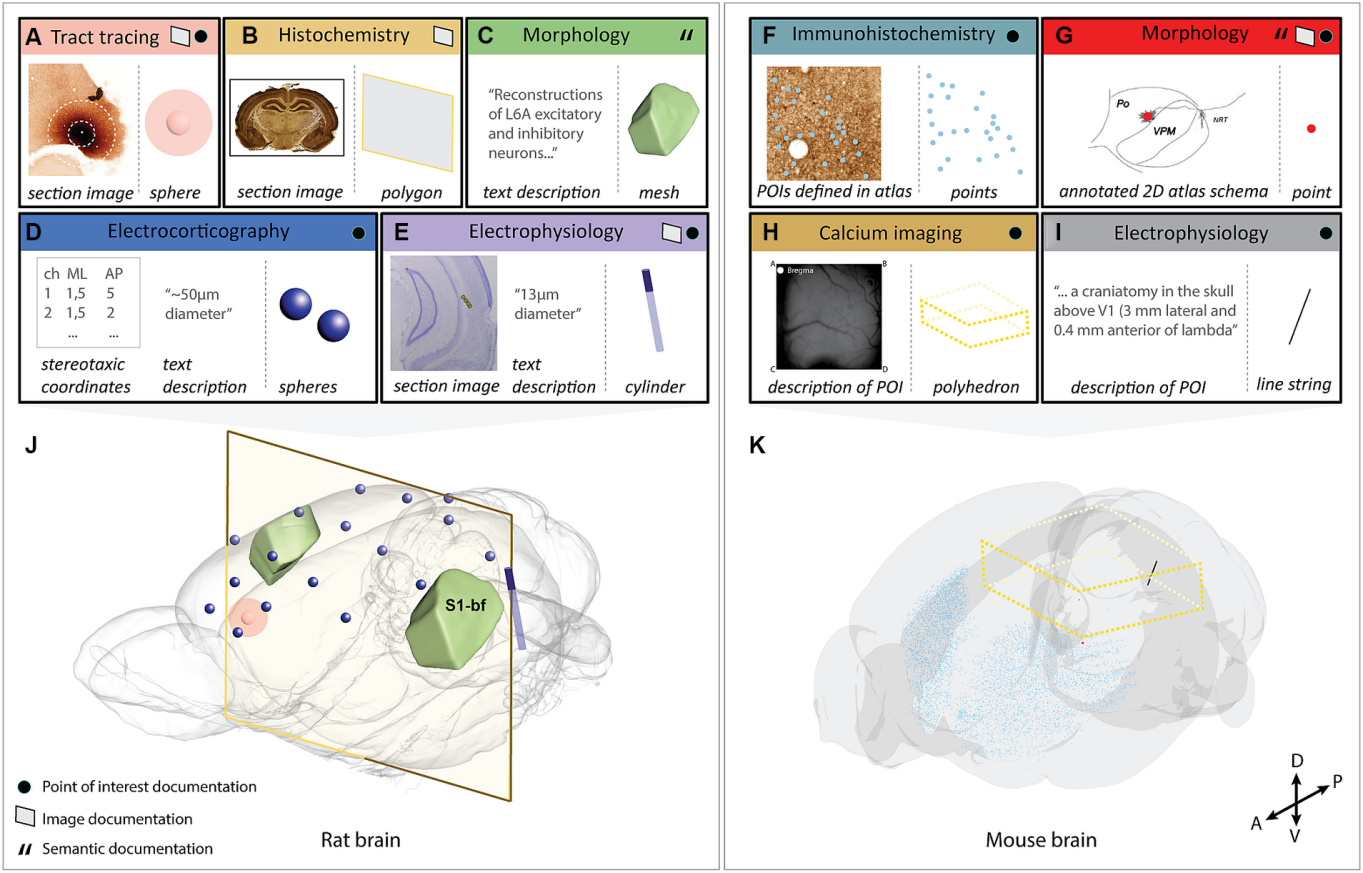
Visualization of the selected use-cases demonstrating the use of the Locare workflow. Use-cases **(A–I)** represented by an input (location documentation) and output (geometric object representation), where the outputs are co-visualized in the respective target atlases **(J)** [Waxholm Space atlas of the Sprague Dawley rat brain or **(K)**; Allen mouse brain atlas Common Coordinate Framework version 3]. **(A)** Image from an anterograde tract tracing experiment showing the injection site placed in the medial orbital area (Kondo et al., 2022). Two spheres represent the position and size of the injection site core and shell, respectively. **(B)** Image from a histochemistry experiment (Blixhavn et al., 2022). A polygon represents the location of the section image. **(C)** Text description from a neuronal reconstruction study (Feldmeyer et al., 2020). An atlas mesh represents the location of the reconstructions. **(D)** Stereotaxic coordinates and radius measurement from electrocorticography experiments (Arena and Storm, 2018; Arena et al., 2019a,b, 2020) using 17 epidural electrodes. A sphere represents the position and extent of each electrode. **(E)** Image from an electrophysiology experiment (Fiorilli et al., 2022) where the electrode track is annotated. A cylinder represents the location of the electrode. **(F)** Image from an immunohistochemistry experiment (Bjerke et al., 2020b) with extracted parvalbumin positive cells annotated. Points represent the extracted cells. **(G)** 2D atlas illustration showing the location of a neuronal reconstruction (García-Amado et al., 2020). A point represents the neuronal soma. **(H)** Descriptions of the field of view used in a calcium imaging experiment (Conti et al., 2019; Resta et al., 2021). A polyhedron represents the field of view. **(I)** A text description of the POI used in an electrophysiology experiment (Schnabel et al., 2020). A line string represents the location of the electrode. **(J)** All use-cases containing data from the rat brain co-visualized in the Waxholm Space atlas of the Sprague Dawley rat brain version 4 (RRID: SCR_017124; Papp et al., 2014; Kleven et al., 2023a; http://www.nitrc.org/projects/whs-sd-atlas/). The coordinates of the objects are opened using MeshView (RRID: SCR_017222) the atlas mesh is opened using Scalable Brain Atlas Composer (Bakker et al., 2015), and objects are overlaid. **(K)** All use-cases containing data from the mouse brain co-visualized in the Allen mouse brain atlas Common Coordinate Framework version 3 (RRID: SCR_020999; Wang et al., 2020). The coordinates of the objects are opened using MeshView and objects are overlaid. S1-bf; primary somatosensory cortex, barrel field.

**Figure 3:**
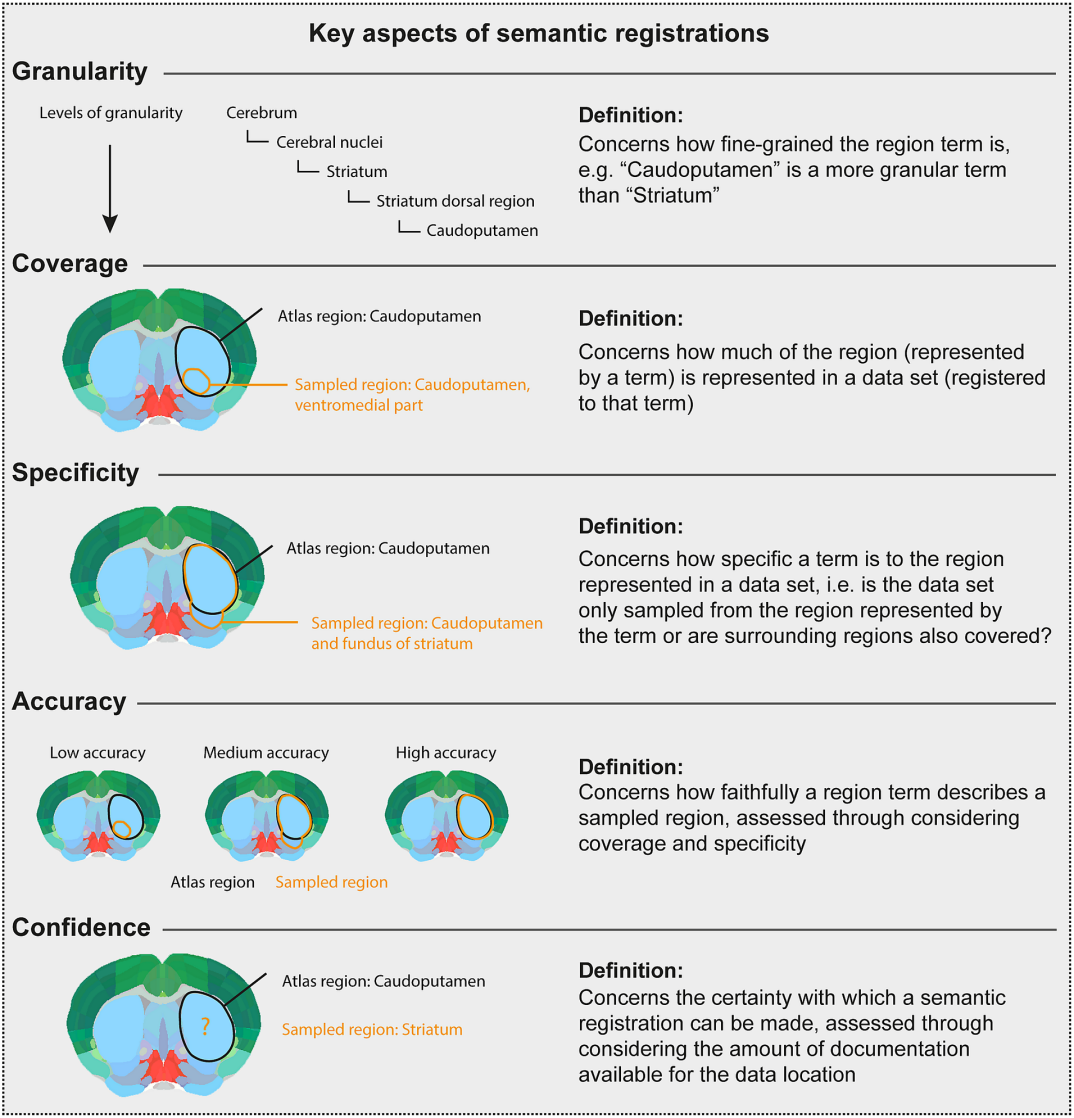
Key aspects of semantic registrations. The Figure [modified from Bjerke (2021)] gives the definition of key considerations when using the semantic route to represent data described using terms from other atlases than the target atlas, or using terms not defined in an atlas. In the atlas plates, black text and lines illustrate a term and an area, respectively, corresponding to a target atlas region. Orange text and lines illustrate a term and an area, respectively, corresponding to a sampled region reported for a data set. Thus, the orange text and lines illustrate the region term and area that should be registered to a target atlas term. The Figure defines and illustrates concepts of granularity, coverage, specificity, accuracy, and confidence.

The Locare workflow takes any information that can be used to define the anatomical location of a sample (e.g., a section or a tissue block) or objects within a sample (e.g., a labeled cell or an electrode) of data as input, independent of methods, data formats, software used for visualization and analysis, and solutions used for sharing the data. This is below referred to as location documentation. Three main categories of location documentation input are distinguished: images, information about POIs, and semantic descriptions. The workflow includes four steps: (1) choosing a target atlas (a 3D brain atlas) and collecting relevant location documentation (Figure 1A); (2) assessing the location documentation (Figure 1B); (3) translating location documentation to spatial metadata in target atlas (Figure 1C); and (4) defining the geometric object representing the location of the data (Figure 1D). A geometric object is a simplified representation of the anatomical location from which the data were derived. If the exact location that the data were derived from cannot be defined, the location can be represented by a geometric object (a mesh) corresponding to an atlas region. The target atlas constitutes the common framework for spatial alignment of data from different sources, enabling meaningful comparisons and integrations.

To exemplify how the output of the workflow can be formatted in a standardized, machine-readable way, we created a collection of JavaScript Object Notation (JSON)^5^ schemas to store the Locare workflow output. The JSON format is widely used due to its suitability for storing semi-structured information, language independence and human readability. Since there are several open standards related to neuroscientific data and geometric representations (such as GeoJSON, NeuroJSON, and openMINDS), we assessed these for inspiration. GeoJSON^6^^,^^7^ is a format for encoding a variety of geographical data structures but is lacking fields to specify the anatomical context for neuroscience data. NeuroJSON^8^ is a JSON-based neuroimaging exchange format. The NeuroJSON JMesh specification can efficiently represent 3D graphical objects, such as shape primitives (spheres, boxes, cylinders, etc.), triangular surfaces or tetrahedral meshes. However, like GeoJSON, the Jmesh specification misses the option to identify the anatomical context. openMINDS (RRID:SCR_ 023173)^9^ is a metadata framework with metadata models composed of schemas that structure information on data within a graph database. Although the schemas of the openMINDS SANDS (RRID:SCR_023498)^10^ metadata model allow for the identification of the anatomical context (semantic and coordinate-based location and relation of data), it is not meant to hold actual (more complex) geometrical data. We chose to base our collection of schemas (LocareJSON) on the GeoJSON standard but extended it to include 3D objects and anatomical context. We defined LocareJSON schemas to the following geometrical objects: point, sphere, line string, cylinder, polygon, polyhedron, and atlas mesh. All LocareJSON schemas define target atlas space through a reference to relevant openMINDS schemas. The Locare atlas mesh schema also defines the relevant atlas mesh through use of openMINDS. For a detailed description of the LocareJSON schemas, see the LocareJSON Github repository (v1.1.1).^11^

### 2.2 Demonstrating the workflow through use-cases

We demonstrate the Locare workflow in a selection of use-cases including heterogeneous data from rat and mouse brains representing each input (location documentation) and output type (geometric objects; Figure 2; Supplementary Table 2). The output resulting from these use-cases were shared in the LocareJSON repository, and as data sets on EBRAINS (Blixhavn et al., 2023a,b,c,d,e,f; Reiten et al., 2023a,b,c). Below, we describe the key tools and processes used to create the use-cases.

We used the Waxholm Space atlas of the Sprague Dawley rat brain (WHS rat brain atlas; RRID: SCR_017124; Papp et al., 2014; Kjonigsen et al., 2015; Osen et al., 2019; Kleven et al., 2023a)^12^ and the Allen mouse brain atlas Common Coordinate Framework (AMBA CCF) version 3 (RRID: SCR_020999; Wang et al., 2020) as our target atlases. For spatial registration, we used the QuickNII (RRID: SCR_016854; Puchades et al., 2019)^13^ and VisuAlign (RRID: SCR_017978)^14^ tools, which come in versions bundled with each of the target atlases.

For extraction of coordinates for a single or a few points of interest, we used the QuickNII mouse-hover function. For more extensive efforts involving numerous points of interest, we used the manual annotation function in the LocaliZoom tool (RRID:SCR_023481),^15^ or the QUINT workflow (Yates et al., 2019; Gurdon et al., 2023)^16^ utilizing QuickNII for registering histological brain section images to the reference atlas followed by tools for extracting (ilastik, RRID:SCR_015246), quantifying, and sorting features according to atlas regions (Groeneboom et al., 2020; RRID: SCR_017183).^17^

To facilitate translation across different atlas terminologies and coordinate systems, we used a set of published data sets containing metadata defining the spatial registration of the rat brain atlas plates of Paxinos and Watson (2018) to the WHS rat brain atlas and the mouse brain atlas plates of Franklin and Paxinos (2007) to the AMBA CCF v3 (Bjerke et al., 2019a,b). These data sets were used to relate stereotaxic landmarks to 3D atlas coordinates, as well as for comparing atlas regions between atlases, as shown in Bjerke et al. (2020a). Since the atlases by Franklin and Paxinos (2007) and Paxinos and Watson (2018) are copyrighted, the data sets do not contain images from these atlases. However, the registration metadata for these data sets can be opened and inspected with locally stored .png images using QuickNII, either to inspect the correspondence of delineations across atlases or to extract WHS rat brain atlas or AMBA CCF v3 coordinates.

To translate spatial metadata from established tools to our example schemas, we created Python scripts for extraction and formatting of (1) QuickNII .json files and (2) Nutil .json coordinate files. The output from QuickNII consists of vectors indicating the position of the 2D image in a 3D atlas (the vector components o, u, v represent the top left corner, and the horizontal and vertical edges of the image, respectively). Coordinates for all four corners can therefore be calculated by addition of vectors. We created scripts^18^ to transform the coordinate output from QuickNII .json files into the LocareJSON schema for polygons. In the Nutil tool, utilized in the QUINT workflow, users can choose whether output coordinates should be given per pixel of an image segmentation, or per centroid of each segmented object. We created scripts^19^ to transform centroid coordinate output from the Nutil tool into the LocareJSON schema for points.

## 3 Results

We here present the Locare workflow and a collection of JSON schemas (LocareJSON) for representing the location of data as geometric objects in 3D atlases. First, we outline the generic steps of the workflow, followed by a description of three different routes for use of the workflow based on the type of location documentation available. Second, we describe the LocareJSON schemas for storing the geometric objects. Lastly, we demonstrate the workflow through nine use-cases representing five different experimental approaches and all the geometrical object types defined by the LocareJSON schemas. Figure 2 gives an overview of the input (location documentation) and output (geometric object representation) for each use-case and visualizes their outputs in their respective 3D target atlases. A summary of details for each use-case is found in Supplementary Table 2.

### 3.1 The Locare workflow

The Locare workflow consists of four steps (Figure 1). The first step (Figure 1A) is to select a target atlas and collect available location documentation, serving as the workflow input. The second step is to assess the available documentation (Figure 1B). The Locare workflow separates location documentation into three main categories: images showing anatomical features, specification of points of interest (POIs), and semantic descriptions. The third step of the workflow (Figure 1C) involves a registration and/or translation process to define coordinates or terms in the target atlas representing the anatomical location of the data set of interest. The fourth and last step (Figure 1D) is to define a geometric object using the appropriate LocareJSON schema. The image and point routes through the workflow yield representations of data location in form of geometric objects, such as points, spheres, line strings, cylinders, polygons, or polyhedrons. The semantic route results in atlas mesh polyhedrons representing an atlas term, which can be used to indicate that data resided somewhere within, or intersecting a given region. The link between the geometric object(s) defined in the Locare workflow and the data set containing the data described in the location documentation is defined in the LocareJSON schema (see section 3.2). Below, we describe the different routes of the workflow in more detail.

#### 3.1.1 The workflow route for points of interest

POIs in a data set can be specified with a broad range of location documentation but are often specified as 2D or 3D points in a coordinate space or image. The POI route through the workflow translates POIs to coordinates in the target atlas and allows users to define geometric objects based on combinations of atlas coordinates. Of the 280 data sets evaluated (Supplementary Table 1), 41 provided documentation of their study target location as POIs.

The Locare workflow distinguishes between three different types of POI documentation (Figure 1B′). First, points may be given as coordinates defined in the target atlas, e.g., coordinates representing features extracted from images, as given for parvalbumin neurons in the data provided by Bjerke et al. (2020b). These coordinates can be used directly to create geometric objects in the target atlas (Figure 1D). Second, points may be specified as coordinates defined in other atlases than the target atlas, for example using coordinates from stereotaxic book atlases (e.g., for the position of implanted electrodes, as provided in use-cases shown in Figures 2D,I). If images from the atlas used to define the POIs are available (Figure 1B′, blue arrow), these can be spatially registered as described in the image route (Figure 1C″, see also section 3.1.2) to enable the translation of the POIs to coordinates in the target atlas. Thirdly, POIs may also represent information about the location of recording sites, images, or other spatial information that can be translated to the target atlas via anatomical landmarks (Figures 2G–I).

When coordinates are defined in the target atlas, they can be used to create all types of geometric objects supported by the LocareJSON schemas. For example, points can be used to represent cell soma positions (Figures 2F,G), a line string could represent the location of an electrode track (Figure 2I), or a polygon could represent the location of a camera field-of-view (the latter may also be extended to a polyhedron to represent the imaging depth captured by the camera; Figure 2H). If the radius for the POI is known, the point object could be replaced by a sphere, or a line string by a cylinder. For example, the location of an electrode track may be represented by a cylinder (Figure 2E), and the location of an injection site core and shell can be represented by a set of spheres with the same centroid point (Figure 2A).

#### 3.1.2 The workflow route for image location documentation

Location documentation in the form of images varies greatly. Images may be magnified microscopy images focusing on specific structures or cover entire brain sections. Image series may contain only a few sections or cover the whole brain (see use-cases shown in Figures 2A,B,F). Image documentation may also be illustrations based on microscopy images, visualizations of reconstructions, or annotations made on atlas plates, as exemplified in Figure 2G. The main process of the image route is to register the images to the target atlas so that coordinate information can be extracted and used to create geometric objects. Of the 280 data sets evaluated in the work with defining this workflow (Supplementary Table 1), 116 provided documentation of their study target location through images.

Images are suitable for spatial registration if they contain specific anatomical features that allow identification of positions in the brain. Thus, in the second step of the workflow route for images (Figure 1C″), the images are examined to see if they meet this criterium. 2D images to be registered should ideally cover whole brain sections, or at least include unique landmarks (Bjerke et al., 2018a) that can be used to determine the angle of sectioning. 3D volumes may cover the whole brain or be partial volumes. Partial 3D volumes to be registered should preferably contain a combination of external and internal anatomical landmarks to allow identification of corresponding locations in an atlas. A range of image registration software are available (Klein et al., 2010; Niedworok et al., 2016; Fürth et al., 2018; Puchades et al., 2019; Tappan et al., 2019), suitable for different types of data and purposes. Further discussions about the choice and application of such tools are provided in reviews by Tyson and Margrie (2022) and Kleven et al. (2023b). Whether or not suitable anatomical landmarks are available for determining the specific anatomical location of a sample should be considered case by case. If the images lack anatomical landmarks, the available information is considered using the semantic route of the workflow.

When registration is performed, the spatial registration output can be used to define geometric objects in the appropriate LocareJSON schema. For 2D images, polygons are used, representing the full plane of the image through defining its four corners (Figure 1D, see also Figures 2A,B). For 3D images, polyhedrons are used, representing the volume through defining the object’s eight corners. For images containing POIs (e.g., annotations of electrode tracks, see Figure 2E), the image route would be used primarily as a mean to define coordinates corresponding to these points. In these cases, it might not be relevant to define geometric objects for the images themselves; instead, the extracted points are taken through the last two steps of the points route (Figures 1C′,D).

#### 3.1.3 The workflow route for semantic location documentation

Semantic location documentation can be any term or description of an anatomical location. This includes a range of documentation types that do not meet the criteria for use in the other routes but still are useful to determine the data location. For example, images that do not contain sufficient anatomical landmarks for spatial registration may be useful for morphological observations of cells of tissue that can be used to determine the anatomical location of data. Semantic location documentation may also include functional characteristics of cells or tissue recorded which could help confirm the location of electrode tracks. The most common form of semantic location documentation, however, is one or more anatomical terms, with or without reference to a brain atlas. Of the 280 data sets evaluated in the work with defining this workflow (Supplementary Table 1), 123 provided documentation of their study target location through semantic descriptions only.

With the semantic route, a brain region term in the target atlas is chosen to represent the location of the data. In the second step of the semantic route (Figure 1B‴), we distinguish between terms defined in the target atlas, terms defined in another atlas, and terms not defined in an atlas. In the third step (Figure 1C‴), data are semantically registered to the target atlas by choosing a final term from the target atlas terminology to represent the data. The approach depends on which type of term was provided. For terms that are already associated to the target atlas, we generally use the term directly as the final term. For terms from other atlases, the registration to the target atlas involves a translation between terminologies, a process depending on defining the correspondence of the region in the other atlas with region(s) in the target atlas. If images of atlas plates from the other atlas are available (Figure 1B‴, yellow arrows), they can be spatially registered as described in the image route (Figure 1C″) and atlas plates can be overlayed with custom atlas overlays from the target atlas. This facilitates translation of terms from the other atlas to the target as described in our previous papers (Bjerke et al., 2020a; Kleven et al., 2023b). If alternative spelling or terms differing from the atlas nomenclature are used, further consideration about underlying definitions and correspondence to the atlas nomenclature is needed. For example, the term “striatum” can be ambiguous, since it may refer to the caudate-putamen (or caudoputamen) alone or the caudate-putamen combined with the nucleus accumbens. Use of parent terms, such as the “substantia nigra” to describe smaller subsets of a region can also introduce ambiguity. In all such cases it is necessary to evaluate available documentation and seek the most precise definition possible.

There are several considerations underpinning the choice of a final term when the initial term comes from another atlas or is not defined in an atlas. This process relies primarily on interpretation of the initial term and documentation by a researcher employing knowledge of neuroanatomy and neuroanatomical atlases, nomenclatures, and conventions. The documentation is evaluated in the choice of final terms, with essential considerations being the specificity, granularity, coverage, and confidence (defined in Figure 3). For example, if a term from another atlas is used, but there is no closely corresponding term in the target atlas, a fine-grained term might be substituted with a coarser term. This would decrease the granularity, but increase the confidence, in the final term. The final term will be chosen from the target atlas terminology, with a corresponding atlas mesh associated to the data set (Figure 1D).

### 3.2 The Locare workflow output: LocareJSON

To exemplify how the geometric object representing the anatomical location of a data element can be formalized in a machine-readable format, we created a collection of JavaScript Object Notation (JSON) schemas, collectively referred to as LocareJSON schemas. These schemas are based on GeoJSON elements and are hosted in the LocareJSON GitHub repository. These LocareJSON schemas provide suitable starting points for researchers who wish to create JSON files storing information about spatial location in the brain. Below we describe the structure and content of the LocareJSON schemas. Each schema consists of a general part (the locareCollection schema) and a part specific to the object it describes (individual object schemas).

The locareCollection schema include the following required properties: versioning of the schema (version), reference to the 3D target atlas (targetAtlas) and one or several persistent links to the original sources for the data (sourcePublication). The targetAtlas is referenced through a link to an openMINDS_SANDS (see text footnote 10) instance (commonCoordinateSpaceVersion). Details about the dimension, resolution, orientation, and origin of target atlas is essential to enable representation of geometric objects in any atlas space, e.g., in an online tool or viewer. The locareCollection schema has two optional properties: related publications (relatedPublications), and online resources (linkedURI, Uniform Resource Identifier). The linkedURI should be used to state an online resource primarily if it links to relevant data already embedded in a tool or viewer (e.g., as for brain section images embedded in the LocaliZoom viewer on EBRAINS, Figure 2A).

The objects supported by LocareJSON (point, sphere, line string, cylinder, polygon, polyhedron, and atlas mesh) are defined in individual schemas. Point representations consist of coordinate triplets, with each triplet defining a specific point in a 3D atlas. Sphere representations build upon point representations and consists of coordinate triplets defining the sphere centroid, with information about radius to create a sphere measured from the centroid. Line string representations consist of two or more coordinate triplets, as a minimum defining the start and end point of a segment. Cylinder representations build upon line string representations with additional information about radius to create a cylinder around the length of the line string. Polygon representations consist of coordinate triplets defining corners of a delimited 2D plane. Polyhedron representations consist of coordinate triplets defining corners of a 3D object (vertices), including information about how vertices create polygons (faces) that can be used to represent 3D objects. Atlas meshes, a unique form of polyhedron, contain the name of a specific term from a 3D atlas, provided by a link to openMINDS_SANDS.

One or several objects can be defined within a locareCollection schema. The schemas for geometric objects include the following required properties: “type,” stating the geometric object type, and “coordinates,” a coordinate list formatted based on the type. The schema for atlas mesh includes the “parcellationEntityVersion,” stating the brain region’s URI. Each object also includes a set of properties pointing to the original data the schema represents. These properties include: the name of the data (“name,” required), clearly directing to a subject, file, or group of files; a description of the data (“description,” required), e.g., “position of cell body”; and a direct link to the data source for the geometric object (“linkedURI”; optional), e.g., the LocaliZoom viewer link for the individual brain section image used to create spheres shown in Figure 2A.

### 3.3 The Locare workflow use-cases

To demonstrate the workflow we applied it to represent the location of data from rats and mice acquired by different methods, including electrophysiology (2 data sets), electrocorticography (1 data set), (immuno-)histochemistry (2 data sets), axonal tract tracing (1 data set), neuronal morphology (2 data sets) and calcium imaging (1 data set), all shown in Figure 2. Technical information about the use-cases is provided in Supplementary Table 2. The rat- and mouse brain data sets were co-visualized in the Waxholm Space atlas of the Sprague Dawley rat brain (Papp et al., 2014; Kjonigsen et al., 2015; Osen et al., 2019; Kleven et al., 2023a) or the Allen mouse brain atlas Common Coordinate Framework (Wang et al., 2020), respectively. For each use-case, we utilized a separate route in the Locare workflow, based on the type of location documentation available, resulting in a LocareJSON schema of which the type depended on the object chosen to represent the data (point, line string, sphere, cylinder, polygon, polyhedron, or atlas mesh). Each use-case is available as a LocareJSON file in the LocareJSON repository and as data sets on EBRAINS, where links to their source data sets and detailed methodological descriptions are also provided.

Figure 2 illustrates how different types of neuroscience data can be represented as geometric objects (Figures 2A–I) that can be co-visualized in an atlas space (Figures 2J,K). The geometric data created as examples are available as derived data sets via EBRAINS (Blixhavn et al., 2023a,b,c,d,e; Reiten et al., 2023a,b,c). The derived data sets are listed in Supplementary Table 2, providing links to LocareJSON files for each use case, as well as to the landing page for each derived data set shown in Figure 2. From the landing page, a data descriptor document is provided, explaining how the geometric data were specified following the Locare workflow, and how the LocareJSON file is organized. These resources provide detailed descriptions of the geometric location data, with suggestions of how they can be visualized. The data coordinates provided can, e.g., be co-visualized in an atlas viewer, such as the MeshView tool, available from EBRAINS.^20^^,^^21^ This tool visualizes brain structures from WHS rat brain atlas and the AMBA CCF mouse brain atlas as geometric meshes and includes a feature for importing point coordinates, such as those provided with our data sets, as shown in Figure 2.

The use-cases demonstrate that the object representation that best represent the data is highly dependent on how the data are made available, and the nature and extent of associated documentation provided with it.

## 4 Discussion

The Locare workflow specifies different ways in which highly variable documentation describing the anatomical location of neuroscience data can be used to create representations of the data as geometric objects in a reference atlas space. The collection of LocareJSON schemas exemplify how such objects can be structured in a machine-readable way. The workflow was established and validated using 280 rat and mouse brain data sets generated using highly different methodologies (Supplementary Table 1). These data sets, shared on the EBRAINS Knowledge Graph between 2018 and 2023, allowed us to categorize the location documentation into three main categories. The geometric object data created for the nine examples used to demonstrate the Locare workflow (Figure 2) are shared as derived data sets on EBRAINS with links to their source data sets (Supplementary Table 2). In our use-cases, coordinates were specified using tools provided via the EBRAINS Research Infrastructure, but numerous other tools for generating 3-D geometric objects and coordinates (see Tyson et al., 2022; Fuglstad et al., 2023) may also be suitable as a starting point to create Locare JSON files. Below, we consider the potential impact, advantages, and limitations of the Locare workflow, including the geometric representations it delivers, and discuss possibilities for utilizing such geometric representations for visualization and spatial queries.

The FAIR guiding principle for data management and stewardship emphasize machine-readability and use of persistent identifiers to optimize reuse of scientific data (Wilkinson et al., 2016). Web-based open data infrastructures, structured metadata, and copyright licenses make data findable, accessible, and re-usable, while use of standardized file formats ensure interoperability of data files with different tools and among similar types of data (Pagano et al., 2013). In the context of the FAIR principles, the Locare workflow allows creation of machine-readable files representing the anatomical location and relevance of different data that otherwise would be difficult to find, access, and compare. By defining geometric objects using atlas-based coordinates, the data representations are spatially integrated and interoperable, in the sense that they can be co-visualized using viewer tools and utilized in various computational processes, including spatial search.

Our use-cases (Figure 2; Supplementary Table 2) show that the usefulness of location documentation depends more on the amount and level of detail of the documentation provided, than the method used to obtain the data. This highlights the need for good reporting practices. It is well established that the amount and consistency of metadata provided with research data varies considerably (see Bjerke et al., 2018a, 2020a), which in turn also contributes to the known problems with low replicability and reproducibility of studies (Goodman et al., 2016; Stupple et al., 2019). The different routes through the Locare workflow accommodates the variability of location documentation typically provided with experimental data sets, thus guiding researchers to define the most specific geometric representations possible with the documentation available for their data sets. In this way, data generated using the same methodology may be represented by different geometric objects when the available metadata differ. The location of a neuronal reconstruction can be defined as a singular point in an atlas (Figure 2G), or only as a mesh representing an entire anatomical subregion when less specific location documentation was provided (Figure 2C). Similarly, a series of histological images registered to an atlas may also be represented in different ways; as polygons representing the locations of sections in atlas space (use-case B), or as a population of points representing specific cellular features extracted from section images (Figure 2F). Improved routines for recording and sharing location documentation for neuroscience data will enable more precise spatial representation of data (Bjerke et al., 2018a; Tyson and Margrie, 2022; Kleven et al., 2023a).

The most detailed and accurate spatial representations of data are achieved by spatial registration of images showing anatomical features. A range of image registration tools are available (Puchades et al., 2019; Tappan et al., 2019; Carey et al., 2023; for review, see Tyson and Margrie, 2022), tailored for different types of 2D or 3D image data, and compatible with different brain atlases. Both manual and automated methods exist for different applications. Scripts are available for converting the output from the spatial registration tool QuickNII to LocareJSON polygon schema (see Figures 2A,B). Similar scripts can readily be adapted to different tools. Once images are spatially registered to an atlas, they can be used to specify points or volumes of interest, such as labeled objects (Figures 2F,G), electrode recording sites (Figure 2C), or tracer injection sites (Figure 2A).

The location of POIs, derived from text descriptions or extracted from atlas-registered images, can result in any geometric object representation. When coordinates for POIs have been extracted, an important consideration is therefore which geometric object would best represent it. There might be several alternatives, as, e.g., in the case of electrode tracks. A point can be used to represent the end or the entry point of the electrode (although the end point is usually most relevant as this is where recordings are made), and a line string may represent both the end and entry point, which would be appropriate when there are recording sites along the track (see Figure 2I, where a linear electrode array with 16 recording sites along the electrode was used). If the radius of the object (e.g., the electrode) is known, points and line strings may alternatively be replaced by spheres and cylinders, by introducing the radius of the object. Determining a radius should be the preferred practice as it benefits both visualization and spatial query purposes. In many cases, however, information about the radius is missing. Whether a best approximation is the better choice must be evaluated on a case-by-case basis.

The Locare workflow defines how the location of disparate neuroscience data can be represented as geometric objects in an atlas space. The workflow was developed using rat and mouse data sets with associated atlases, tools, and resources shared via the EBRAINS Research Infrastructure. The concept of data integration through geometric representations is generic and system independent, and the Locare workflow is therefore in principle applicable for other species for which an open access 3D brain atlas is available, such as, e.g., the zebrafish larvae (Kunst et al., 2019), macaque (Balan et al., 2024), or human brain (Amunts et al., 2020).

With the Locare workflow, we propose a streamlined approach to specify, organize, and store information about anatomical positions in the brain, yielding machine-readable files suitable for search engines, viewers, and other tools. The focus is to represent the location of data in a simplified and standardized format, rather than aiming to integrate the actual data files. We believe this will ensure the relevance of the workflow even when facing new methods, tools, and file formats. Standardized representation of data as geometric objects in 3D coordinate space can be utilized in spatial queries of neuroscience databases. Spatial queries will likely make it easier for researchers to find and reuse relevant data compared to free-text searches, and possibly open for more analytic approaches for re-use of shared data (Cao et al., 2023).

We envision that the Locare workflow can guide researchers describing anatomical locations in their data, and provide a starting point for defining new standards for current and future platforms, thus making neuroscience data more findable, accessible, interoperable and reusable, in accordance with the principles set forward by Wilkinson et al. (2016). Future work will include extension of the concept and workflow to human and non-human primate data and implementation into software for querying and accessing the location and distribution of neuroscience data through atlases.

## Data availability statement

The original contributions presented in the study are included in the article/Supplementary material, further inquiries can be directed to the corresponding author.

## Author contributions

CB: Conceptualization, Data curation, Investigation, Methodology, Validation, Visualization, Writing – original draft. IR: Conceptualization, Data curation, Investigation, Methodology, Validation, Visualization, Writing – review & editing. HK: Data curation, Investigation, Methodology, Validation, Writing – review & editing. MØ: Data curation, Investigation, Methodology, Validation, Writing – review & editing. SY: Investigation, Methodology, Validation, Writing – review & editing. US: Data curation, Investigation, Methodology, Validation, Writing – review & editing. MP: Data curation, Investigation, Methodology, Validation, Writing – review & editing. OS: Data curation, Investigation, Methodology, Project administration, Software, Writing – review & editing. JB: Conceptualization, Funding acquisition, Investigation, Project administration, Resources, Supervision, Validation, Writing – review & editing. IB: Conceptualization, Data curation, Methodology, Supervision, Validation, Visualization, Writing – review & editing. TL: Project administration, Resources, Supervision, Visualization, Writing – review & editing, Conceptualization, Funding acquisition, Methodology.

## Footnotes

^1^https://portal.brain-map.org/

^2^https://www.ebrains.eu/

^3^https://search.kg.ebrains.eu/

^5^https://www.json.org/json-en.html

^6^https://geojson.org/

^7^https://doi.org/10.17487/RFC7946

^8^https://neurojson.org/

^9^https://github.com/HumanBrainProject/openMINDS

^10^https://github.com/HumanBrainProject/openMINDS_SANDS

^11^https://github.com/Neural-Systems-at-UIO/LocareJSON/tree/v1.1.1

^12^http://www.nitrc.org/projects/whs-sd-atlas/

^13^https://quicknii.readthedocs.io

^14^https://visualign.readthedocs.io

^15^https://localizoom.readthedocs.io/en/latest/

^16^https://quint-workflow.readthedocs.io

^17^https://nutil.readthedocs.io

^18^https://github.com/Neural-Systems-at-UIO/LocareJSON/tree/v1.1.1/scripts/quicknii_to_locarePolygons

^19^https://github.com/Neural-Systems-at-UIO/LocareJSON/tree/v1.1.1/scripts/centroids_to_locarePoints

^20^https://www.ebrains.eu/tools/meshview

^21^https://meshview-for-brain-atlases.readthedocs.io/en/latest/index_.html

## References

[ref1] Abrams M. B. Bjaalie J. G. Das S. Egan G. F. Ghosh S. S. Goscinski W. J.. (2022). A standards Organization for Open and FAIR neuroscience: the international Neuroinformatics coordinating facility. Neuroinformatics 20, 25–36. doi: 10.1007/s12021-020-09509-0, PMID: 33506383 PMC9036053

[ref2] Amunts K. Knoll A. Lippert T. Pennartz C. Ryvlin P. Destexhe A.. (2019). The human brain project—synergy between neuroscience, computing, informatics, and brain-inspired technologies. PLoS Biol. 17:e3000344. doi: 10.1371/journal.pbio.3000344, PMID: 31260438 PMC6625714

[ref3] Amunts K. Mohlberg H. Bludau S. Zilles K. (2020). Julich-brain: a 3D probabilistic atlas of the human brain’s cytoarchitecture. Science 369, 988–992. doi: 10.1126/science.abb4588, PMID: 32732281

[ref4] Arena A. Nilsen A. Thon S. Storm J. (2020). Test of consciousness metrics in rodents. [Data set]. EBRAINS. doi: 10.25493/8CQN-Y8S

[ref5] Arena A. Storm J. (2018). Large scale multi-channel EEG in rats. [Data set]. EBRAINS. doi: 10.25493/4SPM-V00

[ref6] Arena A. Thon S. Storm J. (2019a). Mechanistic analysis of ERP in rodents. [Data set]. EBRAINS. doi: 10.25493/5ZJY-PHB

[ref7] Arena A. Thon S. Storm J. (2019b). PCI-like measure in rodents. [Data set]. EBRAINS. doi: 10.25493/S0DM-BK5

[ref8] Ascoli G. Maraver P. Nanda S. Polavaram S. Armañanzas R. (2017). Win–win data sharing in neuroscience. Nat. Methods 14, 112–116. doi: 10.1038/nmeth.4152, PMID: 28139675 PMC5503136

[ref9001] Bakker R. Tiesinga P. Kötter R. (2015). The scalable brain atlas: instant web-based access to public brain atlases and related content Neuroinform 13, 353–366. doi: 10.1007/s12021-014-9258-x PMC4469098 25682754

[ref9] Balan P. Zhu Q. Li X. Niu M. Rapan L. Funck T.. (2024). MEBRAINS 1.0: a new population-based macaque atlas. Imaging. Neuroscience. doi: 10.1162/imag_a_00077 PMC12235559 40800436

[ref10] Bassett D. Sporns O. (2017). Network neuroscience. Nat. Neurosci. 20, 353–364. doi: 10.1038/nn.4502, PMID: 28230844 PMC5485642

[ref9002] Bjerke I. E. (2021). Quantifying cellular parameters across the murine brain: New practices for integrating and analysing neuroscience data using 3D brain atlases. Available at: https://www.duo.uio.no/handle/10852/83758?show=full

[ref11] Bjerke I. Øvsthus M. Andersson K. Blixhavn C. Kleven H. Yates S.. (2018a). Navigating the murine brain: toward best practices for determining and documenting neuroanatomical locations in experimental studies. Front. Neuroanat. 12, 1–15. doi: 10.3389/fnana.2018.00082, PMID: 30450039 PMC6224483

[ref12] Bjerke I. Øvsthus M. Papp E. Yates S. Silvestri L. Fiorilli J.. (2018b). Data integration through brain atlasing: human brain project tools and strategies. Eur. Psychiatry 50, 70–76. doi: 10.1016/j.eurpsy.2018.02.004, PMID: 29519589

[ref13] Bjerke I. Puchades M. Bjaalie J. Leergaard T. (2020a). Database of literature derived cellular measurements from the murine basal ganglia. Sci. Data 7:211. doi: 10.1038/s41597-020-0550-3, PMID: 32632099 PMC7338524

[ref14] Bjerke I. Schlegel U. Puchades M. Bjaalie J. Leergaard T. (2019a). Franklin & Paxinos’ “the mouse brain in stereotaxic coordinates” (3rd edition) spatially registered to the Allen mouse common coordinate framework. [Data set]. EBRAINS. doi: 10.25493/WFCZ-FSN

[ref15] Bjerke I. Schlegel U. Puchades M. Bjaalie J. Leergaard T. (2019b). Paxinos & Watson’s “the rat brain in stereotaxic coordinates” (7th edition) spatially registered to the Waxholm space atlas of the rat brain. [data set]. EBRAINS. doi: 10.25493/APWV-37H

[ref16] Bjerke I. Yates S. Carey H. Bjaalie J. Leergaard T. (2023). Scaling up cell-counting efforts in neuroscience through semi-automated methods. iScience 26:107562. doi: 10.1016/j.isci.2023.107562, PMID: 37636060 PMC10457595

[ref17] Bjerke I. Yates S. Puchades M. Bjaalie J. Leergaard T. (2020b). Brain-wide quantitative data on parvalbumin positive neurons in the mouse. [Data set]. EBRAINS. doi: 10.25493/BT8X-FN9

[ref18] Blixhavn C. Bjerke I. Reiten I. Leergaard T. (2023a). 3D atlas locations of rat brain section images showing nissl bodies, zincergic terminal fields and metal-containing glia. [Data set] EBRAINS. doi: 10.25493/QFFN-H67 PMC10030855 36944675

[ref19] Blixhavn C. Haug F. Kleven H. Puchades M. Bjaalie J. Leergaard T. (2022). Multiplane microscopic atlas of rat brain zincergic terminal fields and metal-containing glia stained with Timm’s sulphide silver method (v1) [data set] EBRAINS. doi: 10.25493/T686-7BX PMC10030855 36944675

[ref20] Blixhavn C. Ovsthus M. Reiten I. Leergaard T. (2023b). 3D atlas location of the field of view of a calcium imaging recording in mouse after stroke (v1). [Data set] EBRAINS. doi: 10.25493/TS1A-K28

[ref21] Blixhavn C. Ovsthus M. Reiten I. Leergaard T. (2023c). 3D atlas locations of mouse thalamocortical projection neuron reconstructions. [Data set] EBRAINS. doi: 10.25493/KGCK-773

[ref22] Blixhavn C. Reiten I. Leergaard T. (2023d). 3D atlas location of electrode recordings in mice during presentation of figure-ground stimuli. [Data set] EBRAINS. doi: 10.25493/H52W-QF4

[ref23] Blixhavn C. Reiten I. Ovsthus M. Bjerke I. Puchades M. Leergaard T. (2023e). 3D atlas locations of epidural electrode EEG recordings in rats. [Data set] EBRAINS. doi: 10.25493/AK1G-WQQ

[ref24] Blixhavn C. Reiten I. Yates S. Leergaard T. (2023f). 3D atlas locations of parvalbumin positive neurons in the adult mouse brain. [Data set] EBRAINS. doi: 10.25493/Q52E-ESE

[ref25] Cao R. Ling Y. Meng J. Jiang A. Luo R. He Q.. (2023). SMDB: a spatial multimodal data browser. Nucleic Acids Res. 51, W553–W559. doi: 10.1093/nar/gkad413, PMID: 37216588 PMC10320082

[ref26] Carey H. Pegios M. Martin L. Saleeba C. Turner A. Everett N.. (2023). DeepSlice: rapid fully automatic registration of mouse brain imaging to a volumetric atlas. Nat. Commun. 14:5884. doi: 10.1038/s41467-023-41645-4, PMID: 37735467 PMC10514056

[ref27] Clarkson M. D. (2016). Representation of anatomy in online atlases and databases: a survey and collection of patterns for interface design. BMC Dev. Biol. 16:18. doi: 10.1186/s12861-016-0116-y, PMID: 27206491 PMC4875762

[ref28] Conti E. Pavone F. Allegra Mascaro A. (2019). Fluorescence cortical recording of mouse activity after stroke. [Data set] EBRAINS. doi: 10.25493/Z9J0-ZZQ

[ref29] Feldmeyer D. Qi G. Yang D. (2020). Morphological data of cortical layer 6 neurons and synaptically coupled neuronal pairs. [Data set] EBRAINS. doi: 10.25493/YMV3-45H

[ref30] Ferguson A. Nielson J. Cragin M. Bandrowski A. Martone M. (2014). Big data from small data: data-sharing in the ‘long tail’ of neuroscience. Nat. Neurosci. 17, 1442–1447. doi: 10.1038/nn.3838, PMID: 25349910 PMC4728080

[ref31] Fiorilli J. Marchesi P. Ruikes T. Buckton R. Quintero M. Reitan I.. (2023). Neural correlates of object identity and reward outcome in the corticohippocampal hierarchy: double dissociation between perirhinal and secondary visual cortex. bioRxiv [Preprint], bioRxiv: 2023.05.24.542117

[ref32] Fiorilli J. Ruikes T. Huis G. Pennartz C. (2022). Sensory, perirhinal and hippocampal tetrode recordings during visual, tactile and visuotactile discrimination task in the freely moving rat (v1). [Data set] EBRAINS. doi: 10.25493/AM91-2D

[ref33] Franklin K. Paxinos G. (2007). The mouse brain in stereotaxic coordinates. 3rd Edn. San Diego: Elsevier Academic Press.

[ref35] Fuglstad J. G. Saldanha P. Paglia J. Whitlock J. R. (2023). Histological E-data registration in rodent brain spaces. Elife 12:e83496. doi: 10.7554/eLife.83496, PMID: 36637157 PMC9904758

[ref36] Fürth D. Vaissière T. Tzortzi O. Xuan Y. Märtin A. Lazaridis I.. (2018). An interactive framework for whole-brain maps at cellular resolution. Nat. Neurosci. 21:895. doi: 10.1038/s41593-017-0058-0, PMID: 29255166

[ref37] García-Amado M. Porrero C. Rubio M. Evangelio M. Clascá F. (2020). 3D reconstruction and measurement of individual thalamocortical projection neuron axons of somatosensory and visual thalamic nuclei. [Data set] EBRAINS. doi: 10.25493/AWS5-MZG

[ref38] Goodman S. Fanelli D. Ioannidis J. (2016). What does research reproducibility mean? Sci. Transl. Med. 8:341ps12. doi: 10.1126/scitranslmed.aaf5027 27252173

[ref39] Groeneboom N. Yates S. Puchades M. Bjaalie J. (2020). Nutil: a pre- and post-processing toolbox for histological rodent brain section images. Front. Neuroinform. 14, 1–9. doi: 10.3389/fninf.2020.00037, PMID: 32973479 PMC7472695

[ref40] Gurdon B. Yates S. Csucs G. Groeneboom N. E. Hadad N. Telpoukhovskaia M.. (2023). Detecting the effect of genetic diversity on brain composition in an Alzheimer’s disease mouse model. *bioRxiv* [preprint]. 10.1038/s42003-024-06242-1 PMC11106287 38769398

[ref41] Jorgenson L. A. Newsome W. T. Anderson D. J. Bargmann C. I. Brown E. N. Deisseroth K.. (2015). The BRAIN initiative: developing technology to catalyse neuroscience discovery. Philos. Trans. R. Soc. B 370:20140164. doi: 10.1098/rstb.2014.0164, PMID: 25823863 PMC4387507

[ref42] Kim Y. Yang G. Pradhan K. Venkataraju K. Bota M. Garcie del Molino L.. (2017). Brain-wide maps reveal stereotyped cell-type-based cortical architecture and subcortical sexual resource brain-wide maps reveal stereotyped cell-type-based cortical architecture and subcortical sexual dimorphism. Cell 171, 456–469. doi: 10.1016/j.cell.2017.09.020 28985566 PMC5870827

[ref43] Kjonigsen L. Lillehaug S. Bjaalie J. Witter M. Leergaard T. (2015). Waxholm space atlas of the rat brain hippocampal region: three-dimensional delineations based on magnetic resonance and diffusion tensor imaging. Neuroimage 108, 441–449. doi: 10.1016/j.neuroimage.2014.12.080, PMID: 25585022

[ref44] Klein S. Staring M. Murphy K. Viergever M. Pluim J. (2010). Elastix: a toolbox for intensity-based medical image registration. IEEE Trans. Med. Imaging 29, 196–205. doi: 10.1109/TMI.2009.2035616 19923044

[ref45] Kleven H. Bjerke I. Clascá F. Groenewegen H. Bjaalie J. Leergaard T. (2023a). Waxholm space atlas of the rat brain: a 3D atlas supporting data analysis and integration. Nat. Methods 20, 1822–1829. doi: 10.1038/s41592-023-02034-3, PMID: 37783883 PMC10630136

[ref46] Kleven H. Reiten I. Blixhavn C. Schlegel U. Øvsthus M. Papp E.. (2023b). A neuroscientist’s guide to using murine brain atlases for efficient analysis and transparent reporting. Front. Neuroinform. 17, 1–8. doi: 10.3389/fninf.2023.1154080, PMID: 36970659 PMC10033636

[ref47] Kondo H. Olsen G. Gianatti M. Monterotti B. Sakshaug T. Witter M. (2022). Anterogradely labeled axonal projections from the orbitofrontal cortex in rat (v1). [Data set] EBRAINS. doi: 10.25493/2MX9-3XF

[ref48] Kunst M. Laurell E. Mokayes N. Kramer A. Kubo F. Fernandes A.. (2019). A cellular-resolution atlas of the larval zebrafish brain. Neuron 103, 21–38.e5. doi: 10.1016/j.neuron.2019.04.034, PMID: 31147152

[ref49] Leergaard T. Bjaalie J. (2022). Atlas-based data integration for mapping the connections and architecture of the brain. Science 378, 488–492. doi: 10.1126/science.abq2594, PMID: 36378966

[ref50] Martone M. Gupta A. Ellisman M. (2004). E-neuroscience: challenges and triumphs in integrating distributed data from molecules to brains. Nat. Neurosci. 7, 467–472. doi: 10.1038/nn1229, PMID: 15114360

[ref52] Niedworok C. Brown A. Cardoso M. Osten P. Ourselin S. Modat M.. (2016). AMAP is a validated pipeline for registration and segmentation of high-resolution mouse brain data. Nat. Commun. 7:11879. doi: 10.1038/ncomms11879, PMID: 27384127 PMC4941048

[ref53] Oh S. Harris J. Ng L. Winslow B. Cain N. Mihalas S.. (2014). A mesoscale connectome of the mouse brain. Nature 508, 207–214. doi: 10.1038/nature13186, PMID: 24695228 PMC5102064

[ref54] Osen K. Imad J. Wennberg A. Papp E. Leergaard T. (2019). Waxholm space atlas of the rat brain auditory system: three-dimensional delineations based on structural and diffusion tensor magnetic resonance imaging. Neuroimage 199, 38–56. doi: 10.1016/j.neuroimage.2019.05.016, PMID: 31100433

[ref55] Pagano P. Candela L. Castelli D. (2013). Data interoperability. Data Sci. J. 12, GRDI19–GRDI25. doi: 10.2481/dsj.GRDI-004

[ref56] Papp E. Leergaard T. Calabrese E. Johnson G. Bjaalie J. (2014). Waxholm space atlas of the Sprague Dawley rat brain. Neuroimage 97, 374–386. doi: 10.1016/j.neuroimage.2014.04.001, PMID: 24726336 PMC4160085

[ref57] Paxinos G. Watson C. (2018). Paxinos and Watson’s The rat brain in stereotaxic coordinates compact. 7th Edn. San Diego: Elsevier Academic Press.

[ref59] Puchades M. Csucs G. Ledergerber D. Leergaard T. Bjaalie J. (2019). Spatial registration of serial microscopic brain images to three-dimensional reference atlases with the QuickNII tool. PloS One 14:e0216796. doi: 10.1371/journal.pone.0216796, PMID: 31141518 PMC6541252

[ref60] Reiten I. Blixhavn C. Bjerke I. Leergaard T. (2023a). 3D atlas location of rat cortical neuron reconstructions. [Data set] EBRAINS. doi: 10.25493/CBTH-1G9

[ref61] Reiten I. Blixhavn C. Leergaard T. (2023b). 3D atlas locations of rat brain injection sites and section images from tract tracing experiments involving the orbitofrontal cortex. [Data set] EBRAINS. doi: 10.25493/R8E4-YKU

[ref62] Reiten I. Ovsthus M. Schlegel U. Blixhavn C. Leergaard T. (2023c). 3D atlas locations of tetrode recordings in rats performing a cross-modal memory recall task. [Data set] EBRAINS. doi: 10.25493/T5VW-SRJ

[ref63] Resta F. Allegra Mascaro A. L. Pavone F. (2021). Study of slow waves (SWs) propagation through wide-field calcium imaging of the right cortical hemisphere of GCaMP6f mice (v2). [Data set] EBRAINS. doi: 10.25493/QFZK-FXS

[ref64] Schnabel U. Lorteije J. Self M. Roelfsema P. (2020). Neuronal activity elicited by figure-ground stimuli in primary visual cortex of the awake mouse. [Data set] EBRAINS. doi: 10.25493/NHHM-1S5 PMC6290763 30542060

[ref65] Sejnowski T. Churchland P. Movshon J. (2014). Putting big data to good use in neuroscience. Nat. Neurosci. 17, 1440–1441. doi: 10.1038/nn.3839, PMID: 25349909 PMC4224030

[ref66] Stupple A. Singerman D. Celi L. (2019). The reproducibility crisis in the age of digital medicine. NPJ Digit. Med. 2:79. doi: 10.1038/s41746-019-0079-z, PMID: 31304352 PMC6550262

[ref67] Tappan S. Eastwood B. O’Connor N. Wang Q. Ng L. Feng D.. (2019). Automatic navigation system for the mouse brain. J. Comp. Neurol. 527, 2200–2211. doi: 10.1002/cne.24635, PMID: 30635922 PMC7561010

[ref68] Tyson A. Margrie T. (2022). Mesoscale microscopy and image analysis tools for understanding the brain. Prog. Biophys. Mol. Biol. 168, 81–93. doi: 10.1016/j.pbiomolbio.2021.06.013, PMID: 34216639 PMC8786668

[ref69] Tyson A. L. Vélez-Fort M. Rousseau C. V. Cossell L. Tsitoura C. Lenzi S. C.. (2022). Accurate determination of marker location within whole-brain microscopy images. Sci. Rep. 12:867. doi: 10.1038/s41598-021-04676-9, PMID: 35042882 PMC8766598

[ref70] Wang Q. Ding S. Li Y. Royall J. Feng D. Lesnar P.. (2020). The Allen mouse brain common coordinate framework: a 3D reference atlas. Cell 181, 936–953.e20. doi: 10.1016/j.cell.2020.04.007, PMID: 32386544 PMC8152789

[ref71] Wilkinson M. Dumontier M. Aalbersberg I. Appleton G. Axton M. Baak A.. (2016). The FAIR guiding principles for scientific data management and stewardship. Sci. Data 3, 160018–160019. doi: 10.1038/sdata.2016.18, PMID: 26978244 PMC4792175

[ref72] Yates S. Groeneboom N. Coello C. Lichtenthaler S. Kuhn P. Demuth H.. (2019). QUINT: workflow for quantification and spatial analysis of features in histological images from rodent brain. Front. Neuroinform. 13, 1–14. doi: 10.3389/fninf.2019.00075, PMID: 31849633 PMC6901597

[ref73] Zaslavsky I. Baldock R. A. Boline J. (2014). Cyberinfrastructure for the digital brain: spatial standards for integrating rodent brain atlases. Front. Neuroinform. 8:74. doi: 10.3389/fninf.2014.00074, PMID: 25309417 PMC4162418

